# Intensive Cultivation of Kiwifruit Alters the Detrital Foodweb and Accelerates Soil C and N Losses

**DOI:** 10.3389/fmicb.2019.00686

**Published:** 2019-04-04

**Authors:** María del Carmen F. Lago, Pedro P. Gallego, María J. I. Briones

**Affiliations:** ^1^Departamento de Ecología y Biología Animal, Vigo, Spain; ^2^Departamento de Biología Vegetal y Ciencia del Suelo, Universidad de Vigo, Vigo, Spain

**Keywords:** *Actinidia deliciosa*, conventional agriculture, integrated practices, organic production, earthworms, microbial biomass, enzymatic activity, nutrient cycling

## Abstract

The detrital food web plays an important role in the functioning of agro-ecosystems due to their positive effect on organic matter transformations and nutrient supply to the growing crops, however, the activities of the organisms involved are strongly influenced by agricultural practices. In NW Spain, commercial Hayward kiwifruit (*Actinidia chinensis var. deliciosa*) is intensively produced using conventional techniques (CONV), however, more sustainable methods, such as integrated (INT) and organic (ORG), have been increasingly adopted to decrease the negative impacts on the environment. We investigated the effects of these agricultural managements on earthworm abundance and functional diversity as well as microbial biomass and enzyme activity and evaluated the potential implications for nutrient retention and runoff in kiwifruit orchards. Our results showed that the CONV soils significantly contained fewer earthworms (ca. 80% less individuals than the INT and ORG systems), with their communities being mainly dominated by small epigeics, but a higher microbial biomass (0.53 ± 0.06 mg C g^-1^ dw soil compared to <0.25 mg C g^-1^ dw soil in INT and ORG), and 20% more activity of the enzymes involved in C (β-glucosidase) and N mineralization (urease). Consequently, more C and N was lost from these soils (on average, >37% more CO_2_, and five times more DIN) than from the less intensively managed soils. In contrast, the INT and ORG systems sustained a more complex and functionally diverse soil food web that lead to higher soil C and N retention. Therefore, agriculture management (i.e., intensive vs. less intensive) and its effects on the structure of the below-ground communities (i.e., microorganisms plus surface detritivores vs. deep burrowers plus geophagous forms) determine the nutrient sink/source function of these agro-ecosystems. These findings highlight the importance of including the contribution of soil biota to soil processes when optimizing fertilization loads and mitigating environmental impacts of agricultural practices.

## Introduction

Currently, 38% of the world’s land surface is cultivated, but this is projected to increase 30% more by 2050 to meet the food demand of a continuous growing human population (FAOSTAT, 2018). This, together with the need to reduce the negative environmental impacts associated to conventional agriculture (Montgomery, 2007; Foley et al., 2011), have prompted the implementation of more cost-efficient sustainable agricultural systems that will ensure high production levels while protecting the environment (van Eekeren et al., 2010; Verhulst et al., 2010; Palm et al., 2014).

Despite China being the largest producer of kiwifruit in the world (1.7 million t y^-1^) accounting for just over half of the global output, many other countries grow the green-fleshed Hayward variety that dominates the food market today (World Kiwifruit Review, 2016). Among them, Spain occupies the 11th position in the list of kiwifruit producing countries (20,884 t y^-1^; MAPAMA, 2018) and more specifically, the Galicia region (NW of Spain) yields 53% of the total national production (11,510 t y^-1^; MAPAMA, 2018). In this Atlantic area, this perennial fruit tree has been traditionally grown using conventional agricultural practices (CONV). However, in the last 25 years, alternative techniques (integrated, INT and organic, ORG) have been slowly adopted to mitigate the harmful effects on the environment.

Soil organic matter (SOM) content and quality is a valuable indicator of soil fertility (Stolze et al., 2000) and can be severely affected by agricultural practices. Crop residues removal and adding large amounts of pesticides, soil pH regulators, mineral fertilizers, etc. result in profound alterations of soil physical and chemical properties, a greater topsoil erosion and biodiversity losses together with increased nutrient leaching, greenhouse gasses emissions, soil acidification and surface water pollution (e.g., Stolze et al., 2000; Kibblewhite et al., 2008; Tsiafouli et al., 2015). In contrast, more sustainable agriculture practices, involving more efficient use of natural resources, favors nutrient retention (Gomiero et al., 2008), and promote soil biological activities (Henneron et al., 2015).

Soil organisms play an important role in the functioning of agroecosystems and among them, earthworms are major indicators of soil quality (Doran and Zeiss, 2000) since they improve soil physical structure and water drainage, facilitate the incorporation of SOM and increase nutrient availability (Blouin et al., 2013; Bertrand et al., 2015). However, in intensively managed agricultural soils, earthworm densities are typically lower compared to uncultivated ecosystems, as a result of the destruction of their habitats, lower food availability and the presence of toxic substances (Postma-Blaauw et al., 2010; Bertrand et al., 2015; Tsiafouli et al., 2015; Briones and Schmidt, 2017). In particular, epigeic (living in the surface horizons) and anecic worms (building vertical burrows) are the most sensitive functional groups to CONV practices (Briones and Schmidt, 2017). The absence of these two ecological groups also have important implications for microbial populations since their casts, either deposited in the lining of their burrow walls or at the opening of their galleries (“middens”; Bohlen et al., 2002), are hotspots for microorganisms. The interaction between earthworms and microbial populations promotes the production of phytohormones and humic substances that stimulate plant growth (Noguera et al., 2010; Puga-Freitas and Blouin, 2015) as well as of those enzymes involved in soil nutrient cycling. Indeed, soil enzyme activities have also been recognized as important indicators of soil fertility, since they have a strong involvement in C, N, and P transformations (Stott et al., 2010; Zhang et al., 2010; Nannipieri et al., 2011; Bowles et al., 2014). Consequently, intensive agricultural managements have long-term effects on soil biota (Johnston et al., 2015), with the detrital food webs becoming less diverse and dominated by smaller sized organisms (Tsiafouli et al., 2015).

Several studies have indicated that ORG agriculture promotes soil biodiversity (e.g., Mäder et al., 2002; Bengtsson et al., 2005; Postma-Blaauw et al., 2010; Flohre et al., 2011; Ponce et al., 2011; Tuck et al., 2014; Henneron et al., 2015). For example, Tuck et al. (2014) estimated that ORG practices increase species richness by approximately 30%, but the responses are highly dependent on the taxonomic group and crop type. Similar studies for INT systems are lacking and consequently, INT agriculture is the poorest studied system in terms of soil biodiversity and soil processes. This is because most research has focused on the comparison between CONV and ORG managements (Randall and James, 2012) and in some cases, researches have combined the results collected from INT systems with those obtained from CONV ones (Mäder et al., 2002). Nevertheless, the little evidence available seems to suggest that these more sustainable managements tend to promote higher biological activities.

Considering the temporal variations in earthworm and microbial communities due to local climatic conditions and soil properties, community responses to agricultural practices requires long-term trials and surveys (Pelosi et al., 2015). For this reason, in this study, we investigated the effects of three long-established (>15 years) management systems (CONV, INT, and ORG) on soil detrital food webs (microbial and earthworm populations) and their activities by taking monthly samples during one full year. We assessed the abundance and functional structure of earthworms, microbial biomass and measured the activity of three extracellular enzymes playing a key role in SOM transformations: (i) urease activity that regulates N supply to plants (Bowles et al., 2014), (ii) β-glucosidase causing the degradation of cellulose (Stott et al., 2010), and (iii) phosphomonoesterase, responsible for P mineralization (Nannipieri et al., 2011). In addition, dehydrogenase activity was also measured since it is an indication of the metabolic state of soil microorganisms (Watts et al., 2010) and it has shown to be affected by farming practices (Liang et al., 2014).

## Materials and Methods

### Kiwifruit Orchards

The investigated systems were located in Tomiño (Galicia, NW Spain: 41°58′20^′′^N, 8°46′34^′′^W). This Atlantic region has a temperate oceanic climate, with a mean annual temperature of 14.6°C and mean annual precipitation of 1627 mm (data collected from the nearest weather station Areas 42^o^1′54^′′^N, 8^o^40′2^′′^W; Meteogalicia, 2015). Temperature and rainfall patterns for the years of study followed a similar trend (mean annual temperature for year 2011 was 15.1 and 14.0°C in 2012; annual precipitation for year 2011 was 1280 and 1502 mm in 2012).

We selected three orchards, where kiwifruit (*Actinidia chinensis* var. *deliciosa* (A. Chev.) A. Chev. cv. “Hayward”) has been produced by either conventional (CONV), integrated (INT), or organic (ORG) farming practices for more than 15 years (Table 1). Despite the difference in sizes, these orchards are representative of the Galician horticultural land use, where smallholdings predominate (usually with one owner), but intensive commercial production is typically managed under cooperatives. Under the most intensive system, high doses of agrochemicals (mineral N fertilizers and two different types of herbicides) are annually applied and the thick tree pruning residues removed from the soil surface to prevent fungal infections. At the INT orchard, herbicide and mineral fertilizers are added annually together with phytorregulators or growth activators [hydrogen cyanamide (CH_2_N_2_) until year 2010 and thereafter calcium cyanamide (Ca(CN)_2_), when the former product was withdrawn from European market to compile with health protection regulations (2008/745/CE)] to synchronize the flowering period between male and female plants (i.e., to shorten the overall flowering time; McPherson et al., 2001). In this treatment, all the coarse plant residues are removed from the soil, whereas the thinnest ones are grounded and deposited onto the soil surface. Finally, at the ORG orchard, no herbicides are added and nutrient additions consist of legally approved ORG fertilizers and homemade compost made of pruning residues, poultry slurry and pine needles. Details of orchard extensions and other external inputs are shown in Table 1.

**Table 1:** Management history and physico-chemical properties measured at the conventional (CONV), integrated (INT), and organic (ORG) kiwifruit orchards.

	CONV	INT	ORG
Cultivated Area (ha)	20.9	11.8	0.5
Orchard starting year	1987	1986	2000
Previous land use	*Pinus pinaster* forest	*Pinus pinaster* forest	Nursery, greenhouse crops and fodder production
Herbicides	Ammonium glufosinate (C_5_H_15_N_2_O_4_P)15%, Dose: 3–5 L ha^-1^, once a year; Terbuthylazine (C_9_H_16_ClN_5_) 50%, Dose: 2 L ha^-1^, once a year	Glyphosate ((N-(phosphonomethyl) glycine)), Dose: 3 L ha^-1^, twice a year	No applied
Inorganic N inputs (kg ha^-1^)	93.4	60.0	No applied
Organic N inputs (kg ha^-1^)	No applied	No applied	170
P inputs (kg ha^-1^)	57.0	25.0	8.8
K inputs (kg ha^-1^)	108.7	140.0	338.2
Ca inputs (kg ha^-1^)	360.9	35.0	40.4
Mg inputs (kg ha^-1^)	6.9	20.0	6.8

Bulk density (Kg m^-3^ dw)^∗^	876 ± 50ab	857 ± 50a	1018 ± 27 b
Texture	Sandy-loam	Sandy-loam	Sandy-loam
C (%)^∗^	5.9 ± 0.3 a	4.8 ± 0.2 ab	3.6 ± 0.1 b
N (%)^∗^	0.50 ± 0.03 a	0.47 ± 0.03 a	0.43 ± 0.03 a
C/N^∗^	13.2 ± 0.6 a	11.3 ± 0.5 b	9.5 ± 0.4 c
Soil pH^∗^	5.99 ± 0.09 a	6.17 ± 0.09 b	6.47 ± 0.02 b
Soil temperature (^o^C)^∗^	13.4 ± 1.1a	13.5 ± 1.1b	14.2 ± 1.2c
Soil moisture (%)^∗^	29.2 ± 1.5a	22.8 ± 0.7b	22.1 ± 0.8b


*^∗^Different letters show significant differences between managements (Tukey’s HSD test; p < 0.05).*

### Field Sampling and Sample Analyses

At each studied orchard (CONV, INT, and ORG), earthworms and soil samples were collected monthly, from December 2011 to December 2012. Care was taken to include the spatial variability present at each farm and accordingly, we sampled different areas each time. Because kiwifruit vines are trained to grow onto T-bar trellises in rows 4 m apart, samples were taken in between wine rows to avoid any damages to the vines. Therefore, on each sampling occasion three soil quadrats (50 × 50 × 20 cm) were randomly selected on different locations and were hand sorted in the field to collect the earthworms. Abundance of total earthworms and individual ecological categories were obtained and expressed as individuals m^-2^. In addition, six replicate soil cores were randomly taken using PVC cylinders (10 cm diam. × 20 cm deep) for microbial and soil analyses. Half of the total number of these soil samples (3) were used for measuring soil respiration and DOC, DON, DIN and DIP concentrations in the leachates, whereas the other half number of cores were used for soil moisture content (SWC), microbial biomass (C_mic_) and soil enzymatic activity determinations (13 sampling times × 3 orchards × 3 replicates = 78 samples for each determination).

Soil respiration was measured from each intact soil cores using an infra-red gas analyzer (ADC MGA-3000). On every sampling occasion, each soil core was placed into an air-tight glass jar (1000 mL), where it was fluxed with CO_2_-free air for 120 s. CO_2_ concentration was immediately measured (t_0_) and again after 30 min incubation in darkness (t_30_) to enable the calculation of flux rates (t_30_–t_0_). Respiration rates were expressed as μg CO_2_-C g^-1^ dw soil day^-1^.

After these measurements were taken, each individual core was leached with 250 mL of distilled water added to the soil core surface, left it drain under gravity and then re-applied to the core surface twice to ensure the equilibrium of the mineralized nutrients between the soil and the leachates (Anderson and Ineson, 1982). Leachates were then filtered (7–9 μm, FilterLabR, Ref. 1252) and subsamples of 50 mL were used to obtain the concentrations of: (i) dissolved organic C (DOC), using a continuous flow autonalyzer (Sievers Innovox TOC Analyzer); (ii) total dissolved N (TDN); (iii) dissolved inorganic N (DIN = ammonium (N-NH_4_^+^) + nitrites (N-NO_2_^-^) + nitrates (N-NO_3_^-^)) by colorimetry using a Bran + Luebbe-AA3 continuous flow autoanalyzer (Bran + Luebbe, Norderstedt, Germany), and (iv) dissolved inorganic P (DIP), colorimetrically using a spectrophotometer (Thermo Scientific Evolution 60S). DON concentrations in the soil solution were obtained as the difference between TDN and DIN. DOC, DON, DIN, and DIP results were expressed as mg L^-1^ except for those of N-NO_2_^-^, which were expressed in μg L^-1^.

Soil moisture was measured gravimetrically after oven drying the fresh soil sample (2–4 g) at 105^o^C to constant weight and the results were expressed as percentage. One data logger (HOBO H08) was inserted in the soil of each orchard at 10 cm depth to register soil temperatures during the investigated period.

Total soil C and N contents were determined after combustion of air dried and sieved samples (<2 mm) using a LECO elemental analyzer (CN-2000, LECO Corp., St. Joseph, MI, United States), with the results being expressed as percentage; the C/N ratio was also calculated from these data.

Microbial biomass (C_mic_) was estimated by means of the fumigation-extraction method (Vance et al., 1987), with the fumigated and non-fumigated extracts being analyzed with a continuous flow autoanalyzer (Sievers Innovox TOC Analyzer) and expressed as mg g^-1^ dw soil. The potential activity of four soil enzymes was measured colorimetrically: (i) β-glucosidase (BG) following the method Trasar-Cepeda et al. (2003) and the results were expressed in μmol of para-nitrophenol (*p*NP) released g^-1^ dw soil h^-1^; (ii) Urease (UR) according to the method of Kandeler et al. (1999) and the results were expressed in μmol NH_4_^+^-N g^-1^ dw soil h^-1^; (iii) Acid phosphomonoesterase (APM) following Trasar-Cepeda et al. (1985, 2003) and the results were expressed in μmol *p*NP released g^-1^ dw soil h^-1^, and (iv) Dehydrogenase activity (DHA) was quantified according to the method described by von Mersi and Schinner (1991) and the results were expressed in μmol p-iodonitrotetrazolium formazan (INTF) g^-1^ dw soil h^-1^.

### Calculations and Statistical Analyses

In order to evaluate the effect of the CONV agricultural practices on the soil food web the V index (Wardle and Parkinson, 1991 in Wardle, 1995) was calculated:

$$V=\frac{2MCONV}{MCONV+MREF}-1$$

where MCONV and MREF are either the percentages of basal resources (C, N, and P), the abundance or biomass of soil organisms or the rates of enzymatic activity under the most intensive CONV system and the reference (REF) treatment (here INT and ORG treatments), respectively. The index V ranges from -1 (extreme inhibition of the CONV system) to +1 (maximum stimulation of the selected response variables by the CONV system) and with 0 indicating no differences. The degree of these responses is further classified into six categories: extreme inhibition: V < -0.67; moderate inhibition -0.67 < V < -0.33; mild inhibition -0.33 < V < 0; mild stimulation 0 < V < 0.33; moderate stimulation 0.33 < V < 0.67 and extreme stimulation V > 0.67 (Wardle, 1995).

Data were log-transformed prior to performing the parametric analyses to meet the normality and homogeneity criteria. However, even after transformation, some response variables still failed to meet the homogeneity criterion. Since Lindman (1974) concluded that the *F* statistic is robust against violations of this assumption, we used the log-transformed values in all statistical analyses to keep consistency in the choice of data transformations throughout the study (Onofri et al., 2010).

Thereafter, repeated measures ANOVA was used to test for significant differences between treatments, with agricultural practices as fixed factor and sampling time as a repeated factor. Separation of means was determined using Tukey’s HSD (honestly significant difference) test (α = 0.05). Soil temperature data were analyzed using one way ANOVA for unbalanced designs since the number of observations was unequal among treatments.

Detrended Canonical Correspondence Analysis (DCCA) was also used to relate soil chemical and biological properties, followed by the Monte Carlo test (*p* < 0.05) to determine the significance of the canonical axes.

DCCA analyses were performed using the CANOCO software for Windows v4.5 (ter Braak and Smilauer, 2002), whereas the ANOVA analyses were performed using STATISTICA v10 (StatSoft, Inc., 2011).

## Results

### Effects of Agricultural Practices on Soil Community Structure and Enzymatic Activities

**FIGURE 1:**
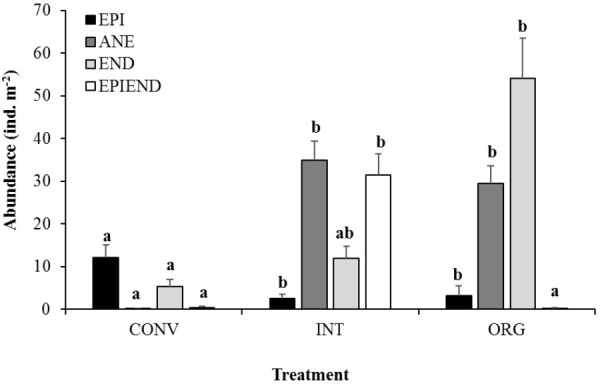
Average abundance of epigeic (EPI), anecic (ANE), endogeic (END), and epiendogeic earthworms (EPIEND) collected at the conventional (CONV), integrated (INT), and organic (ORG) kiwifruit orchards. Error bars are standard errors. Different letters indicate significant differences between agricultural managements per ecological grouping (Tukey’s HSD test: pEPI = 0.0018, pANE < 0.00001, pEND = 0.0119, pEPIEND = 0.0001).

Agricultural management had a significant effect on earthworm populations (*p* < 0.05), with the lowest abundances of these organisms being recorded in the CONV orchard (78 and 79% less individuals than in the INT and ORG orchards, respectively). Importantly, the structure of earthworm community showed important alterations with agriculture intensification (Figure 1), and in particular, those species belonging to the anecic group were drastically reduced in the most intensive treatment (CONV), with the majority of the individuals being small epigeic worms (Figure 1). In contrast, the less intensively managed treatments supported significantly higher numbers of anecic worms, but also other functional groups, such as epiendogeic (INT) or endogeic worms (ORG), were better represented in these soils than in the CONV ones (Figure 1).

**FIGURE 2:**
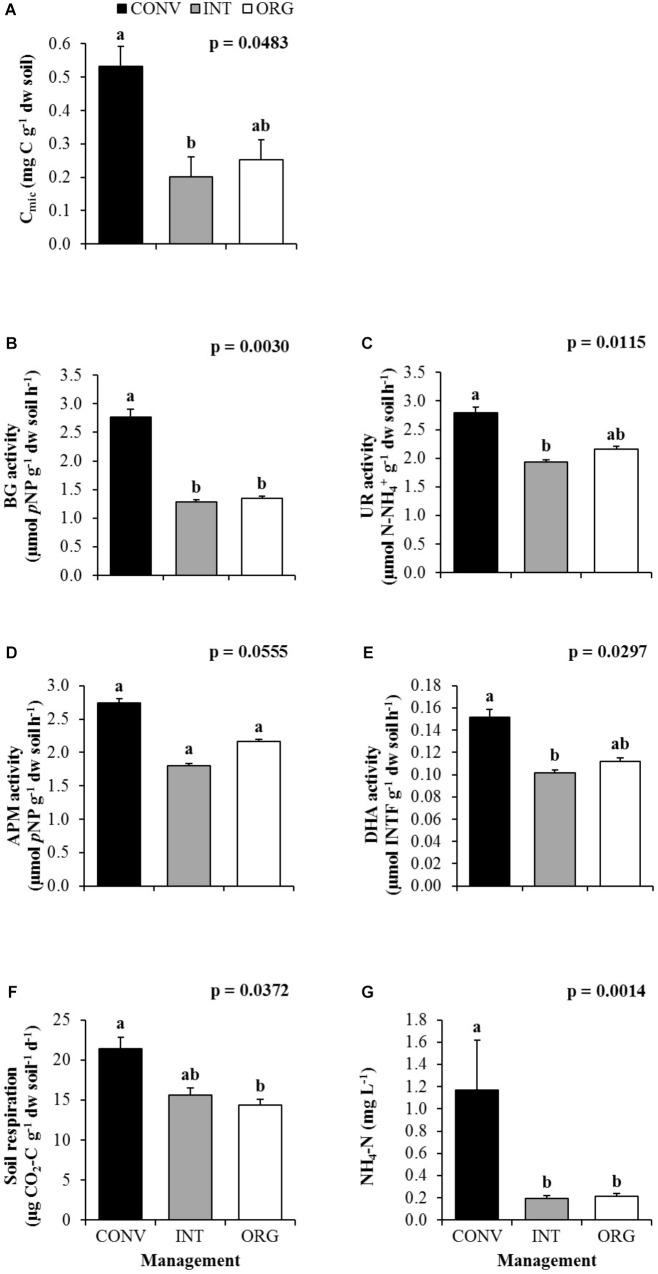
Average values of **(A)** microbial biomass (C_mic_), **(B)** β-glucosidase (BG), **(C)** Urease (UR), **(D)** Acid phosphomonoesterase (APM), **(E)** Dehydrogenase (DHA), **(F)** soil respiration and **(G)** ammonium (NH_4_^+^-N) measured at the conventional (CONV), integrated (INT), and organic (ORG) kiwifruit orchards. Error bars are standard errors. Different letters indicate significant differences between agricultural managements (Tukey’s HSD test; *p* < 0.05).

Similarly, agricultural management also had a strong influence on soil microorganisms (*p* < 0.05). However, unlike earthworms, the CONV treatment clearly favored microbial populations and a higher values of microbial biomass was measured under the most intensive management compared to the INT and ORG systems (Figure 2A). Consequently, CONV soils also showed the highest activity of all four enzymes investigated (Figure 2B–E), but in particular of BG involved in the plant cellulose degradation (Figure 2B).

### Effects of Agricultural Practices on C, N, and P Fluxes

Because of a higher microbial activity, a higher metabolic rate was also measured at the CONV treatment (Figure 2F), with 37 and 49% more CO_2_ being emitted from these soils to the atmosphere when compared to the less intensively managed orchards (INT and ORG, respectively). In contrast, despite the higher amounts of DOC, DON, and DIP released into the soil solution when the soils were more intensively managed, no significant effect of agricultural management was detected, with the exception of DIN in the form of NH_4_^+^-N. This was the result of significantly higher concentrations of ammonium being lost from the CONV soils (five times more than from the INT and ORG agro-ecosystems; Figure 2G).

### Effects of Intensive Agricultural Practices on the Detrital Soil Food Web: Implications for Soil Processes

**FIGURE 3:**
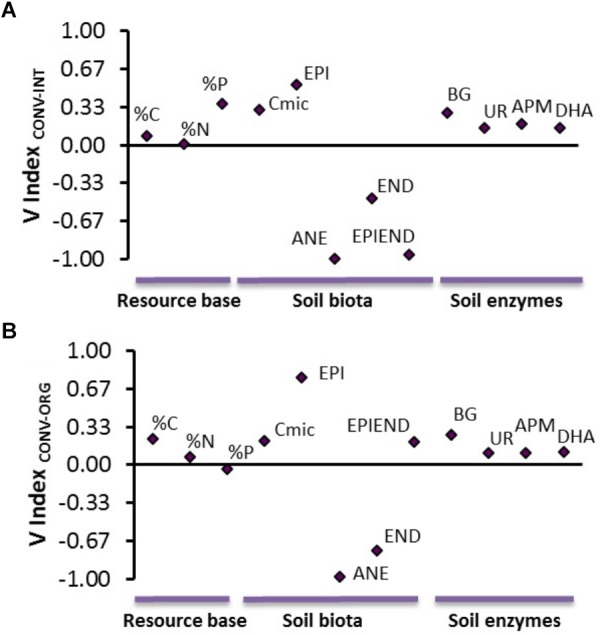
Average values of the V index for **(A)** CONV vs. INT and **(B)** CONV vs. ORG comparisons including the soil resource bases (% C, % N, and % P), soil biota and microbial enzyme activities. Abbreviations: EPI, epigeics; ANE, anecics; END, endogeics; EPIEND, epiendogeics; Cmic, microbial biomass; BG, β-Glucosidase; UR, urease; DHA, dehydrogenase; APM, acid phosphomonoesterase; %C, carbon content; %N, nitrogen content; %P, phosphorous content.

The results from the V index indicated that earthworms were the most sensitive soil organisms to the intensive production of kiwifruits (Figure 3). In particular, anecic and epiendogeic earthworms were the two groups most severely affected, by showing extreme inhibition responses when compared to their populations at the INT and ORG systems (Figure 3). Conversely, the CONV management promoted epigeic species (extreme/mild stimulations compared to the INT and ORG systems, respectively) and microbial populations (both microbial biomass and soil enzymes were moderately stimulated; Figure 3). These positive effects of CONV management on these small-sized organisms were associated with a higher resource base availability in the CONV soil compared to the other two treatments (Figure 3).

**FIGURE 4:**
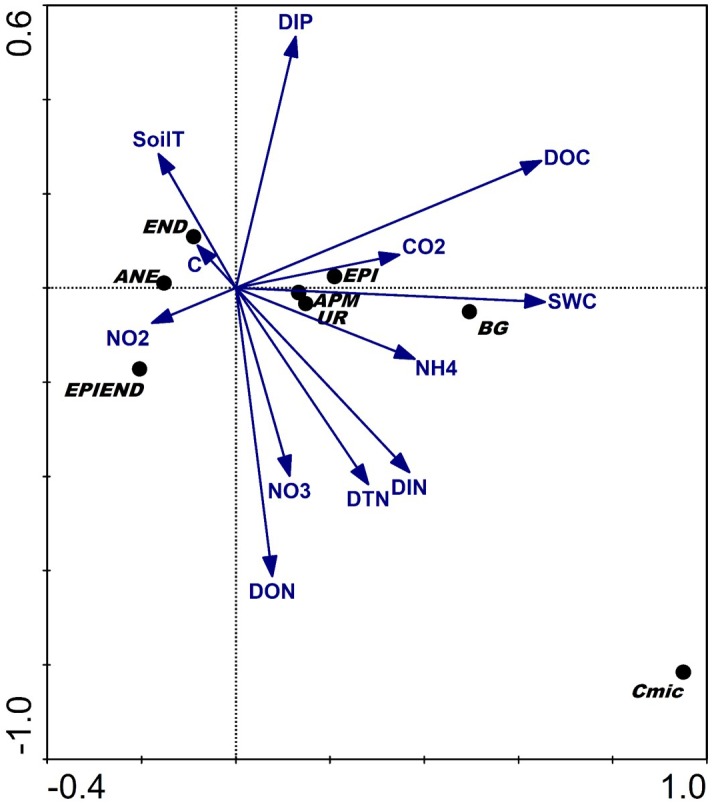
Detrended Canonical Correspondence Analysis (DCCA) biplot of the soil biological and chemical characteristics measured at the investigated orchards based on the first two axes. Abbreviations: EPI, epigeics; ANE, anecics; END, endogeics; EPIEND, epiendogeics; Cmic, microbial biomass; BG, β-Glucosidase; UR, urease; APM, acid phosphomonoesterase; C, carbon content; CO_2_, soil respiration; DOC, dissolved organic carbon; NH_4_, dissolved ammonium; NO_2_, dissolved nitrites; NO_3_, dissolved nitrates; DIN, dissolved inorganic nitrogen; DON, dissolved organic nitrogen; TDN, total dissolved nitrogen; DIP, dissolved inorganic phosphorous; SWC, soil water content; soilT, soil temperature.

The multivariate ordination analysis (DCCA) biplot based on the first two axes, and explaining 77.9% of the variance (and with the first canonical axis being significant: Table 2), confirmed that the amounts of C, N, P released from these agricultural soils together with SWC were the most important variables explaining the observed variation in biological properties of these soils under different agricultural managements. Accordingly, the higher microbial, enzymatic activities and epigeic earthworm abundance were positively related to greater C and N losses in those soils with higher water contents (Figure 4). In contrast, the higher abundance of anecic and endogeic earthworms were positively related to accelerated leaching of inorganic P (Figure 4). These results suggest a stronger influence of microbial and epigeic earthworm activities on C and N mineralization, whereas anecic and endogeic worms seem to play a more important role in P mobilization.

**Table 2:** DCCA ordination summary for the first four canonical axes and results from the Monte- Carlo permutation test.

Axes	I	II	III	IV	Total inertia
Eigenvalues	0.223	0.043	0.025	0.009	1.370
Soil biology-environment correlations	0.660	0.428	0.332	0.268	
Cumulative percentage variance of soil biology data	16.3	19.5	21.3	21.9	
Cumulative percentage variance of soil biology-environment relation	65.2	77.9	85.3	87.8	

Sum of all canonical eigenvalues 0.342					
Summary of Monte Carlo test (499 permutations under full model)					
Test of significance of first canonical axis: eigenvalue = 0.223					
*F*-ratio = 20.041					
*P*-value = 0.0020					
Test of significance of all canonical axes: Trace = 0.342					
*F*-ratio = 2.856					
*P*-value = 0.0020					


## Discussion

Several studies have confirmed that intensive agricultural practices have negative effects on soil biodiversity, making soil food webs less diverse and composed of smaller bodied organisms (Tsiafouli et al., 2015). Accordingly, in this study, earthworm densities were drastically reduced in the CONV system and, in particular, those of the bigger-sized anecic worms. The herbicides commonly used in this type of intensive cultivations are known to compromise the survival of these surface feeders (Parfitt et al., 2005; Carey et al., 2009; Postma-Blaauw et al., 2010; Fründ et al., 2011). Indeed, the dominant anecic species at the study site, *Lumbricus friendi*, is known to be extremely sensitive to these chemical inputs, with their abundances becoming more severely reduced than those of other species (Schreck et al., 2012).

Our results also confirmed that epigeic worms have a poor representation in these orchard soils, which coincides with previous observations in other fruit orchards in France (Feldmanstern, 2013). However, the greater abundance of these surface dwellers observed in the CONV management could be related to their short life cycles (r-strategists). Their high reproductive rates allows for a rapid restoration of their population sizes after environmental disturbances (Bouché, 1977). In addition, their smaller size also makes them more resistant to land use intensification (Tsiafouli et al., 2015; Briones and Schmidt, 2017). Numbers of endogeic earthworms were also very low in the CONV orchards, probably because no additional organic matter amendments were added and the prunning residues were removed from the soil surface. Organic matter input is an important factor controlling the population sizes of this ecological group and mineral fertilizers are insufficient to sustain their populations (Marhan and Scheu, 2005; Simonsen et al., 2010; Feldmanstern, 2013).

Increased earthworm abundance reduced microbial populations, which suggest that that either earthworms feed on microorganisms and/or they competed for similar organic matter pools in soil (Marhan and Scheu, 2005). Consequently, the CONV soils with the fewest earthworms had the highest microbial biomass and potential activity of the four enzymes studied. However, it is generally assumed that ORG systems (or those under minimal anthropogenic activity) have a greater enzymatic activity (Aon and Colaneri, 2001; Oehl et al., 2004; Benitez et al., 2006; Melero et al., 2006; Mijangos et al., 2006; Badalucco et al., 2010; Paz-Ferreiro et al., 2011; Aparna et al., 2014; Sofo et al., 2014). This is because SOM provides a greater number of microhabitats for microorganisms (Paz-Ferreiro et al., 2011) and tends to be higher in these organically managed systems. Therefore, other factors might play a more crucial role in regulating microbial activities, such as the amount and origin of SOM (Marhan and Scheu, 2005). Furthermore, a recent meta-analysis study (Lori et al., 2017) concluded that reported differences in microbial size and activity between CONV and ORG systems vary as a function of land use (arable, orchards, and grassland), plant life cycle (annual and perennial) and climatic zone and thereby, offering a plausible explanation for the conflicting results. For example, the greater C pools measured in the CONV soils contrast with other studies that indicate that ORG farming increases top soil carbon stocks (e.g., Gattinger et al., 2012). Although some large-scale studies have concluded that soil C sequestration dynamics is not determined by age since land use conversion (Deng et al., 2016), the fact that the previous use of our ORG system was extractive (Table 1) has probably resulted in the depletion of soil C pools and hence, less food for microorganisms.

Importantly, the significantly higher activity of the BG enzyme in the CONV system, with a crucial role in the C cycle (Tejada et al., 2009), also explains why the highest soil respiration rates were also measured in these intensively managed soils, and suggest that SOM was more rapidly mineralized under CONV practices. Furthermore, the lower number of earthworms present in the CONV soils and the less microbial grazing stated earlier have also been suggested as important factors stimulating soil respiration (Uvarov, 2016).

Not only more carbon was lost from the CONV treatment, but also nitrogen, in the form of ammonium (the final product of the UR activity; Krajewska, 2009; Das and Varma, 2011), was leached in greater quantities from these soils, which also showed the highest nitrification rates. The CONV orchard annually receives high doses of inorganic nitrogen, however, if the crop demand is less than the amounts added, there is a potential risk of increased leaching rates of this nutrient (Bationo, 2004) and thus, could lead to water pollution. Consequently, the nutrient losses might not compensate the investments in mineral fertilizers (Riley et al., 2001).

A different situation was observed in the ORG system where P was the main mobilised element into the soil solution (DIP). Soil fauna has an important role in the mobilization of soil P (Suthar, 2007; Chapuis-Lardy et al., 2011; Bender and Heijden, 2014) and in particular, endogeic worms have shown to have a predominant role in its release (Simonsen et al., 2010; Coulis et al., 2014). This ecological grouping clearly dominated the earthworm communities in the ORG soils and were positively related with increased concentrations of water soluble inorganic P in the soil solution.

Interestingly, the INT orchard, which aims to be half way between the CONV and ORG systems, in the sense of minimizing the use of pesticides and fertilizers through better targeting and integration with cultural control of weeds, pests and diseases (Boatman et al., 2007), was clearly more beneficial to the detrital food web and retained more C and N than the most intensive management. In addition, no significant differences, neither in microbial biomass nor enzyme activities, were observed between INT and ORG treatments, which is in agreement with previous results (Von Lützow et al., 2002), and suggest that INT agriculture represents a good agriculture practice to enhance soil fertility.

## Conclusion

Although the three managed orchards were not truly replicated (regarding the number of sampling units), which could pose some limitations to our overall interpretations and potential extrapolations to other areas, they are representative of Galician kiwifruit production systems, located in the same climatic area and for which we have a lot of background information. This has that allowed us testing whether a single factor (management) can result in significant differences in the biological and physico-chemical properties. Accordingly, the results from this study clearly evidence that intensively managed kiwifruit orchards (CONV) sustain less complex soil food webs, primarily consisting of surface epigeic worms and microorganisms, capable of a rapid mineralization of SOM and consequently, these systems became net sources of CO_2_, DOC, and DIN. In contrast, the less intensively managed soils (INT and ORG), supported a more diverse soil food web, composed of bigger bodied organisms and in particular, higher numbers of anecic worms, which have a more crucial role in improving soil structure and SOM incorporation (Briones and Schmidt, 2017) and ultimately, higher C and N retention. However, in the case of the ORG treatment, more inorganic P was released from these soils, through the stimulating action of endogeic earthworms. Phosphorous is a non-renewable resource and is often a limiting factor for crop yield. The leaching of phosphate to the water systems can result in environmental problems, which has prompted the adoption of measures to improve plant phosphorus use efficiency (e.g., Veneklaas et al., 2012). Therefore, future sustainable policies should not only include measures to promote more functionally richer soil communities, but also improve soil physical and chemical parameters, according to the crop type and climate zone to avoid nutrient losses, greenhouse emissions and to ensure the delivery of ecosystem services.

## Author Contributions

ML collected and analyzed the data. MB and PG designed the experimental procedures. All authors equally contributed to the discussions for data interpretation and drawing the conclusions.

## Conflict of Interest Statement

The authors declare that the research was conducted in the absence of any commercial or financial relationships that could be construed as a potential conflict of interest.
